# A Biomimetic Microfluidic Tumor Microenvironment Platform Mimicking the EPR Effect for Rapid Screening of Drug Delivery Systems

**DOI:** 10.1038/s41598-017-09815-9

**Published:** 2017-08-24

**Authors:** Yuan Tang, Fariborz Soroush, Joel B. Sheffield, Bin Wang, Balabhaskar Prabhakarpandian, Mohammad F. Kiani

**Affiliations:** 1 Department of Mechanical Engineering, Temple University, Philadelphia, PA 19122 USA; 2 Department of Biology, Temple University, Philadelphia, PA 19122 USA; 3 Department of Biomedical Engineering, Widener University, Chester, PA 19013 USA; 4 Biomedical Technology, CFD Research Corporation, Huntsville, AL 35806 USA; 5 Department of Radiation Oncology, Lewis Katz School of Medicine, Temple University, Philadelphia, PA 19140 USA

## Abstract

Real-time monitoring of tumor drug delivery *in vivo* is a daunting challenge due to the heterogeneity and complexity of the tumor microenvironment. In this study, we developed a biomimetic microfluidic tumor microenvironment (bMTM) comprising co-culture of tumor and endothelial cells in a 3D environment. The platform consists of a vascular compartment featuring a network of vessels cultured with endothelial cells forming a complete lumen under shear flow in communication with 3D solid tumors cultured in a tumor compartment. Endothelial cell permeability to both small dye molecules and large liposomal drug carriers were quantified using fluorescence microscopy. Endothelial cell intercellular junction formation was characterized by immunostaining. Endothelial cell permeability significantly increased in the presence of either tumor cell conditioned media (TCM) or tumor cells. The magnitude of this increase in permeability was significantly higher in the presence of metastatic breast tumor cells as compared to non-metastatic ones. Immunostaining revealed impaired endothelial cell-cell junctions in the presence of either metastatic TCM or metastatic tumor cells. Our findings indicate that the bMTM platform mimics the tumor microenvironment including the EPR effect. This platform has a significant potential in applications such as cell-cell/cell-drug carrier interaction studies and rapid screening of cancer drug therapeutics/carriers.

## Introduction

Tumor drug delivery is a complex phenomenon affected by several elements including physico-chemical properties of drug and/or delivery vehicle. A better understanding of the tumor microenvironment is critical to the development of successful targeted therapeutics. In fact, despite the success of the targeting concepts in clinical trials, e.g. imatinib mesylate (Gleevec®), gefitinib (Iressa®), trastuzumab (Herceptin®), and cetuximab (C225, Erbitux®), high efficacy drug delivery to cancer remains a daunting challenge primarily due to the heterogeneity and complexity of the tumor microenvironment^1^.

Similar to normal tissue microenvironment, cells in tumor microenvironment (including tumor and stromal cells, fibroblasts, and immune cells) are embedded in the extracellular matrix surrounded by blood vessels which supply nutrition and oxygen^2^. On the other hand, tumor microenvironment possesses some unique features including leakiness and discontinuity of tumor endothelial cells in the vasculature, poor oxygenation, low pH and high interstitial pressure^3^. Because of these differences, selective targeting to tumor microenvironment is possible by the enhanced permeation and retention (EPR) effect^4, 5^. EPR effect is one of the most widely used modalities for passive targeting of macromolecules to solid tumor^4^, although the significance of the EPR effect, especially in human tumors has been questioned^6, 7^. The difference in porosity and pore size of tumor vasculature endothelium has made selective targeting possible for many types of nanocarriers. Therefore, reproducing the EPR effect *in vitro* is one of the important factors for representing the tumor microenvironment.

Traditionally, tumor drug discovery relies heavily on murine models to screen for efficacy before progressing to clinical trials^8^. However, strong concerns regarding genomic and phenotypic correspondence between human and murine models and their relevance to human disease have recently been expressed by the scientific community^9, 10^. Overall, murine models are expensive and require skilled personnel, not to mention the physiological differences between murine and human tissues. In contrast, *in vitro* models are cost-effective means for pre-clinical studies and screenings of novel therapeutics. Many 3D *in vitro* tumor models, such as the widely used spheroid hanging drop method, comprise of cancer cells and have the potential to better represent the *in vivo* conditions^11^. However, these static spheroid models do not account for transport across the vascular endothelium and do not reproduce the complex network structure and fluid shear observed in the *in vivo* tumor microenvironment. Furthermore, they rely exclusively on diffusion of the drug molecules to permeate the tumor, and do not allow real-time visualization to study the delivery of the drug or the drug carrier. In general, static *in vitro* models of tumor drug delivery show poor correlation with *in vivo* performance^12^.

Recent research has focused on the development of microfluidic devices to study cell-based phenomena^13, 14^. However, traditional linear channels are typically two-dimensional in nature and are not well-suited for the study of tumor drug delivery. Early stage microfluidic devices and tissue engineering techniques for fabricating 3D constructs that mimic *in vivo* cellular interactions lack the tumor microenvironment (comprising of tumor and vascular cells) and the ability to study real-time interactions and visualizations of the drugs within the 3D cellular environment^15^. In the past few years, more advanced devices featuring co-cultured tumor and endothelial cells for studying tumor angiogenesis/metastasis have been widely reported^16–19^. However, since these devices are designed to study cell migration, they usually employ several parallel straight micro-channels for easy access and imaging but are not suitable for the study of drug delivery/drug carrier extravasation behaviors observed under the complex tumor vasculature. On the other hand, traditional transwell and many current biomimetic tumor-on-a-chip models^20–22^ aiming at characterizing tumor drug transport usually feature a more complex and realistic vessel network. However, these systems cannot offer real-time observation of tumor-endothelium interaction/drug molecule diffusion/drug carrier extravasation due to the stacked architecture of compartments.

In order to better understand the impact of heterogeneity and complexity of the tumor microenvironment, we have developed a microfluidics-based platform^12^ for real-time monitoring of drug delivery in a geometrically realistic environment encompassing (a) circulation in the vessels, (b) transport across the vessel walls, and (c) delivery to the tumors across the interstitial space. The goal of the current study is to establish an *in vitro* tumor microenvironment that approximates *in vivo* tumor barrier characteristics to reproduce the enhanced permeability and retention (EPR) effect with permeability values similar to those reported *in vivo*. This novel *in vitro* biomimetic microfluidic tumor microenvironment (bMTM) platform featuring co-culturing of human breast cancer cells and human breast tumor associated endothelial cells (HBTAEC), not only replicates the 3D morphology of cellular architecture observed *in vivo*, but also mimics the tumor environment using specific tumor derived matrices from tumor tissue constructs. Highly metastatic cancer cells can down-regulate the expression of endothelial cell-cell adhesion molecules, alter endothelial cell biomechanical properties and even break down endothelial barrier function^23^. In this study, we hypothesize that endothelial cells co-cultured with metastatic MDA-MB-231 tumor cells in the bMTM platform will cause the vascular channels to become leakier than those co-cultured with non-metastatic MCF-7 tumor cells.

## Results

### Endothelial cells and tumor cells were successfully co-cultured in the bMTM

**Figure 1:**
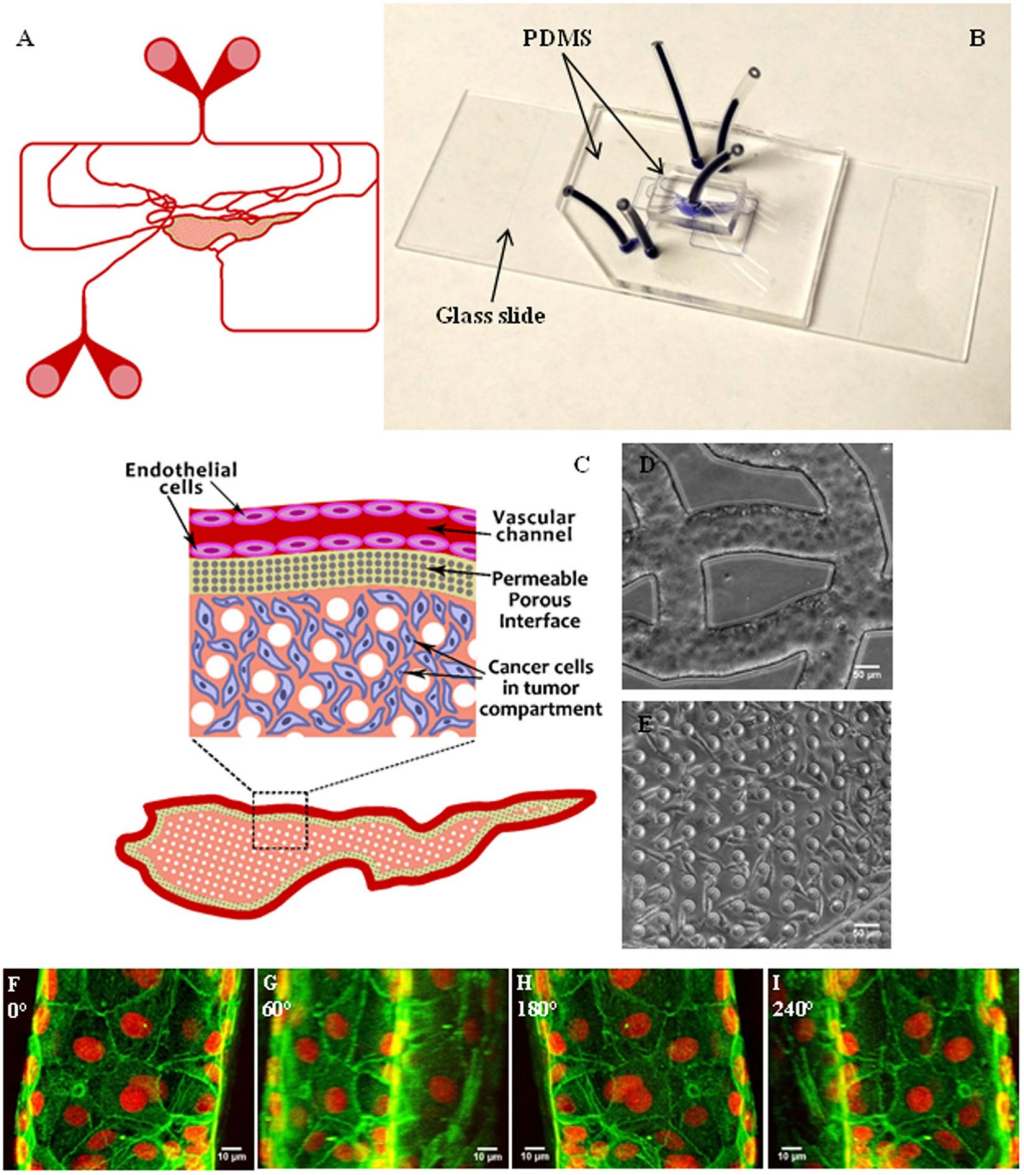
Schematic of the bMTM (**A**) with magnified view of the vascular compartment, vascular-tumor compartment interface and the tumor compartment (**B**). Optical image of the bMTM (**C**) with HBTAEC cultured in the vascular compartment (**D**) and MDA-MB-231 cultured in the tumor compartment (**E**). HBTAEC cultured under flow in the vascular compartment of bMTM form a complete lumen as shown with 3D reconstruction of confocal images of HBTAEC cultured in bMTM stained with f-actin (green) and Draq5 (red) after 4 days in culture maintained under flow of 0.05 μl/min (**F**–**I**); images are shown with a Y-axis rotation of 0, 60, 180 and 240 degrees in (**F**,**G**,**H** and **I**) respectively.

A soft lithography based process was used to construct bMTM with both vascular and tumor compartments separated by a tightly packed cylindrical micro-pillar array. The geometry of vascular channels (Fig. 1A) and dimensions that closely approximate the size and morphology of microvessels *in vivo* permit the bMTM platform to maintain physiologically relevant shear flow conditions in vascular channels for endothelial cell growth. To allow for real-time monitoring of tumor-endothelial cell interaction in both tumor and vascular compartments (e.g. the assessment of HBTAEC permeability in the bMTM), the platform is constructed from optically clear PDMS assembled on a microscope slide (Fig. 1B). Biochemical and cellular communication between the two compartments is achieved through the porous interface (Fig. 1C) provided by the micro-pillar arrays. Co-culturing of tumor cells and endothelial cells were achieved by first establishing endothelial cells in the vascular compartment (Fig. 1D) followed by culture of tumor cells in the tumor compartment (Fig. 1E). HBTAEC cultured under flow formed a complete 3D lumen in the vascular channels (Fig. 1F–I, Video S1), which mimics the normal endothelial cell lining observed *in vivo*.

### Permeability of endothelial cells to small dye molecules increased in the presence of metastatic breast tumor cells

**Figure 2:**
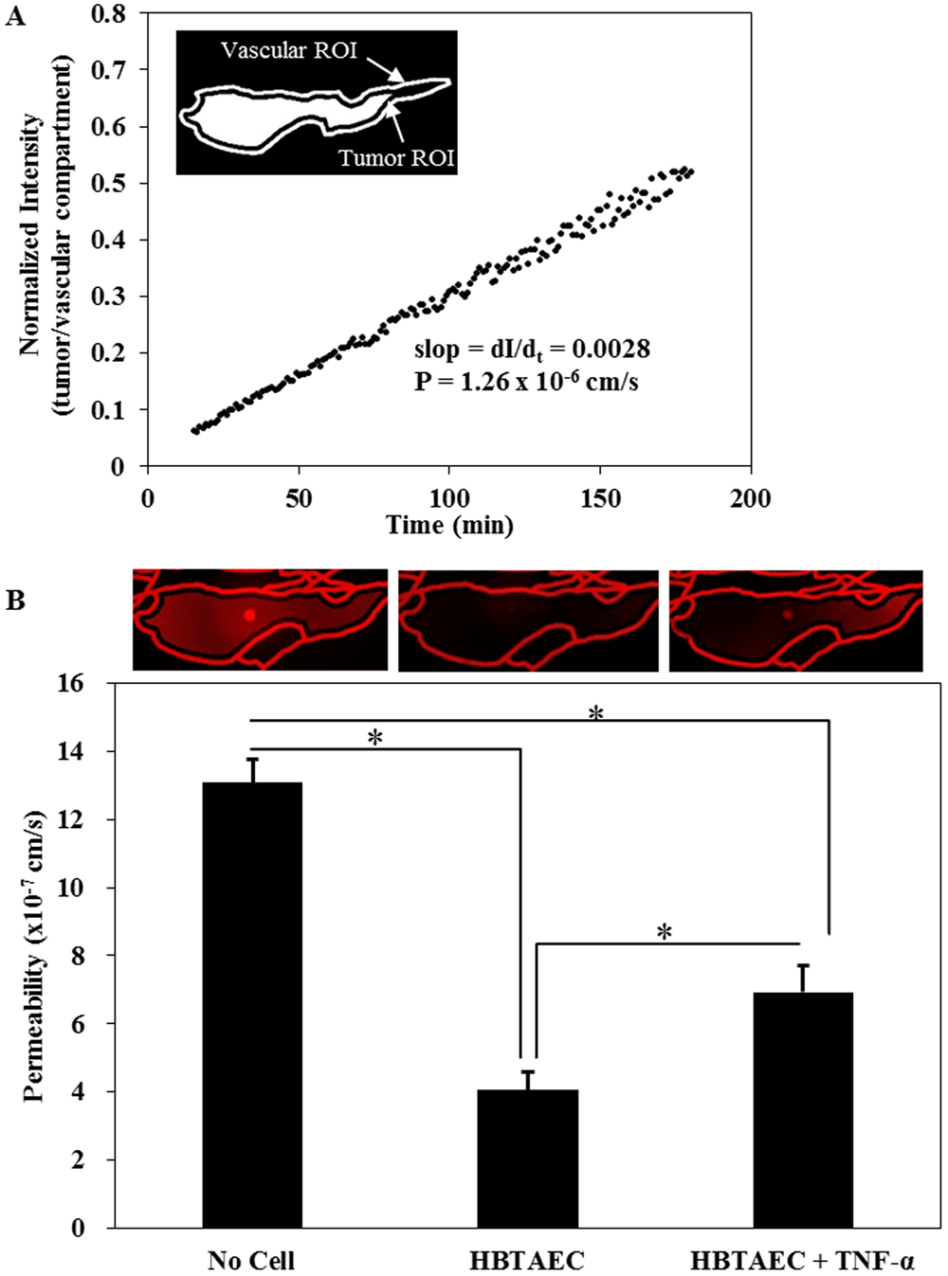
Culturing tumor endothelial cell (HBTAEC) in the vascular compartment for 48 h significantly decreased permeability compared to bMTM with no cells. Permeability of the fluorescent 40 kD dextran from vascular compartment to the tumor compartment in a cell-free bMTM were quantified by normalizing the fluorescent intensity in the tumor to the vascular compartment while fluorescent dextrans diffuse from the vascular compartment to the tumor compartment (panel A). Permeability increased when endothelial cells were treated with TNF-α but was still significantly lower than in cell-free bMTM (panel B). Data are presented as mean ± SEM (n = 3). *Significant difference by *ANOVA*.

Since the bMTM is a dynamic *in vitro* model of tumor microenvironment for real-time monitoring and direct measurement of permeability across endothelium using microscopic methods, we first optimized the techniques for quantifying permeability in the bMTM. Permeation of the fluorescent 40 kD dextran from the vascular compartment to the tumor compartment either with or without the presence of tumor was characterized by measuring fluorescence of dextran injected into the vascular compartment at a flow rate of 1 μl/min (i.e. shear rate 0–90 s^−1^) similar to our previous studies^24^. Quantification of permeability was performed by calculating the average intensity of fluorescent dextran in the entire tumor compartment and normalizing it to the maximum intensity of fluorescent dextran in the vascular compartment. The results of a typical experiment with no cells shown in Fig. 2A indicate that the normalized intensity increases linearly with time in the tumor compartment. The slope of the line (dI/d_t_) was used to calculate the permeability of HBTAEC to the fluorescent dextran. Culturing HBTAEC in the vascular compartment of the bMTM significantly decreased its permeability to the fluorescent dextran when compared to bMTM with no cells (Fig. 2B). Permeability increased when endothelial cells were treated with TNF-α but was still significantly lower than bMTM with no cells.

**Figure 3:**
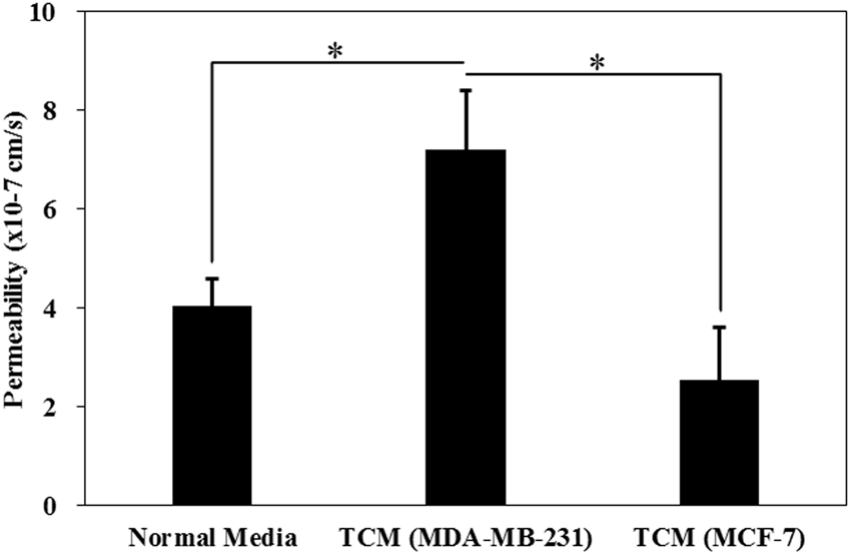
Tumor conditioned media (TCM) (48 h treatment) from highly metastatic MDA-MB-231 tumor cells significantly increased HBTAEC permeability to 40 kD Texas Red dextran compared to treatment with TCM from less metastatic MCF-7 tumor cells or when cultured with normal media. Data are presented as mean ± SEM (n = 3). *Significant difference by *ANOVA*.

The effect of tumor conditioned media (TCM) on HBTAEC permeability in the bMTM was characterized by mixing fresh HBTAEC culture media at 50/50 ratio with either MDA-MB-231 or MCF-7 conditioned media which was injected into the vascular compartment of bMTM for 48 hours. The results shown in Fig. 3 indicate a significantly increased permeability of HBTAECs when they were treated with TCM from highly metastatic MDA-MB-231 tumor cells as compared to TCM from non-metastatic MCF-7 tumor cells or cultured with normal media.

**Figure 4:**
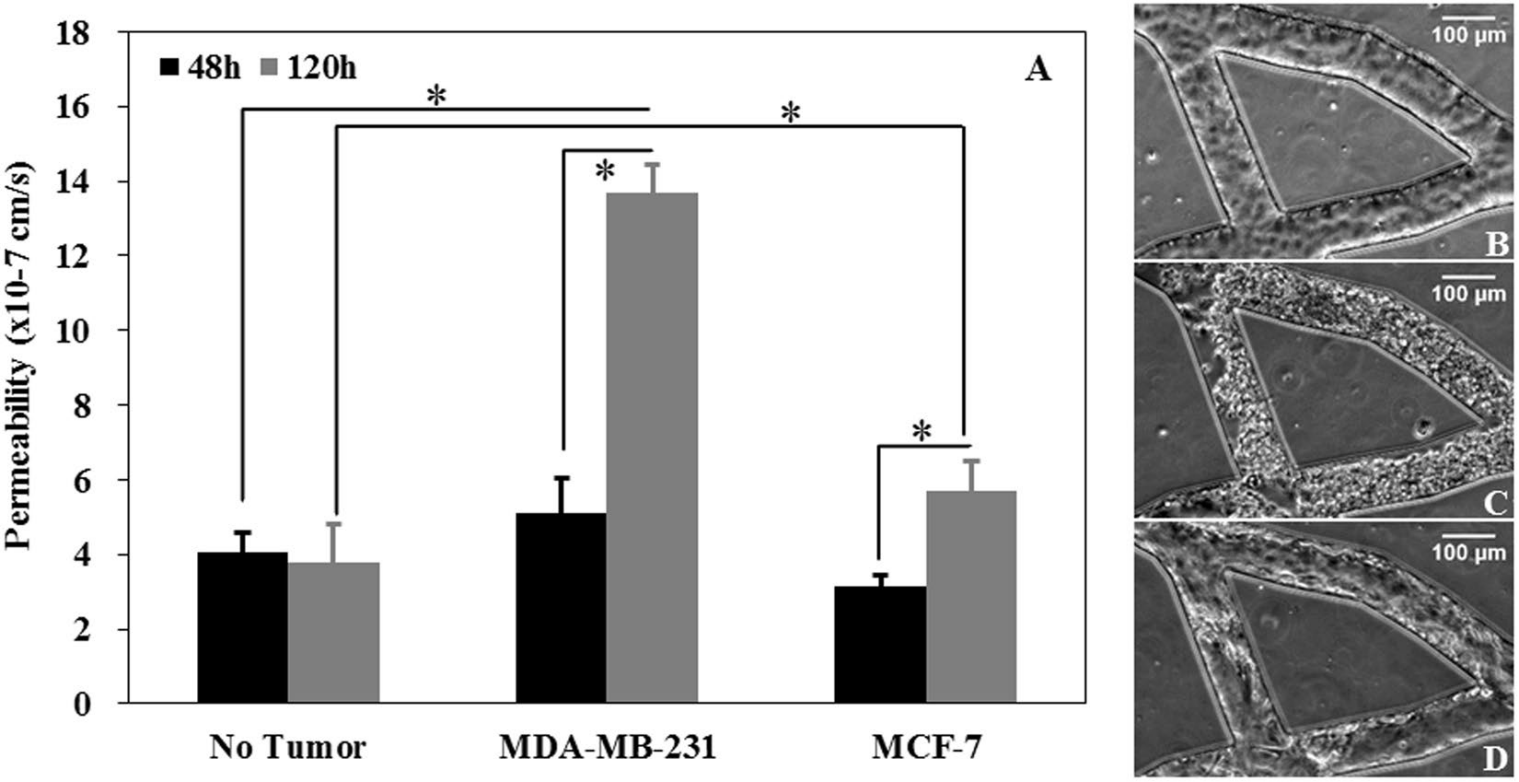
Permeability of HBTAEC increased significantly with increasing co-culture duration with highly metastatic MDA-MB-231 cells (panel A) and was accompanied by morphological changes in endothelial cells (panels B–D). HBTAEC cultured without tumor cells showed no change in morphology for 120 h (panel B) but endothelial cell morphology changed significantly when co-cultured with MDA-MB-231 cells for 120 h (panel C). HBTAEC co-cultured with MCF-7 cells for 120 h showed fewer morphological changes (panel D). Data are presented as mean ± SEM (n = 3). *Significant difference by two-way *ANOVA*.

Permeability of HBTAEC in co-culture with tumor cells in bMTM was measured to determine how presence of tumor cells impact the EPR effect. Changes in permeability of HBTAEC in co-culture with tumor cells (Fig. 4A) are consistent with morphological changes in HBTAEC (Fig. 4B,C and D). These changes could not be easily observed during the first 48 hours of co-culture, but with prolonged co-culture, the endothelial permeability increased significantly as measured by quantitative permeability assay (Fig. 4A). The effect of co-culture on permeability and HBTAEC morphology was more prominent in highly metastatic MDA-MB-231 tumor cells as compared to less metastatic MCF-7 tumor cells. Although HBTAEC permeability increased with increased MCF-7 co-culture time, this increase was not significant when compared to HBTAEC cultured alone. The permeability of endothelial cells without tumor co-culture did not change during the 5 days of experimentation.

### Extravasation of liposomal drug carriers from vascular to tumor compartment increased in the presence of metastatic breast tumor cells

**Figure 5:**
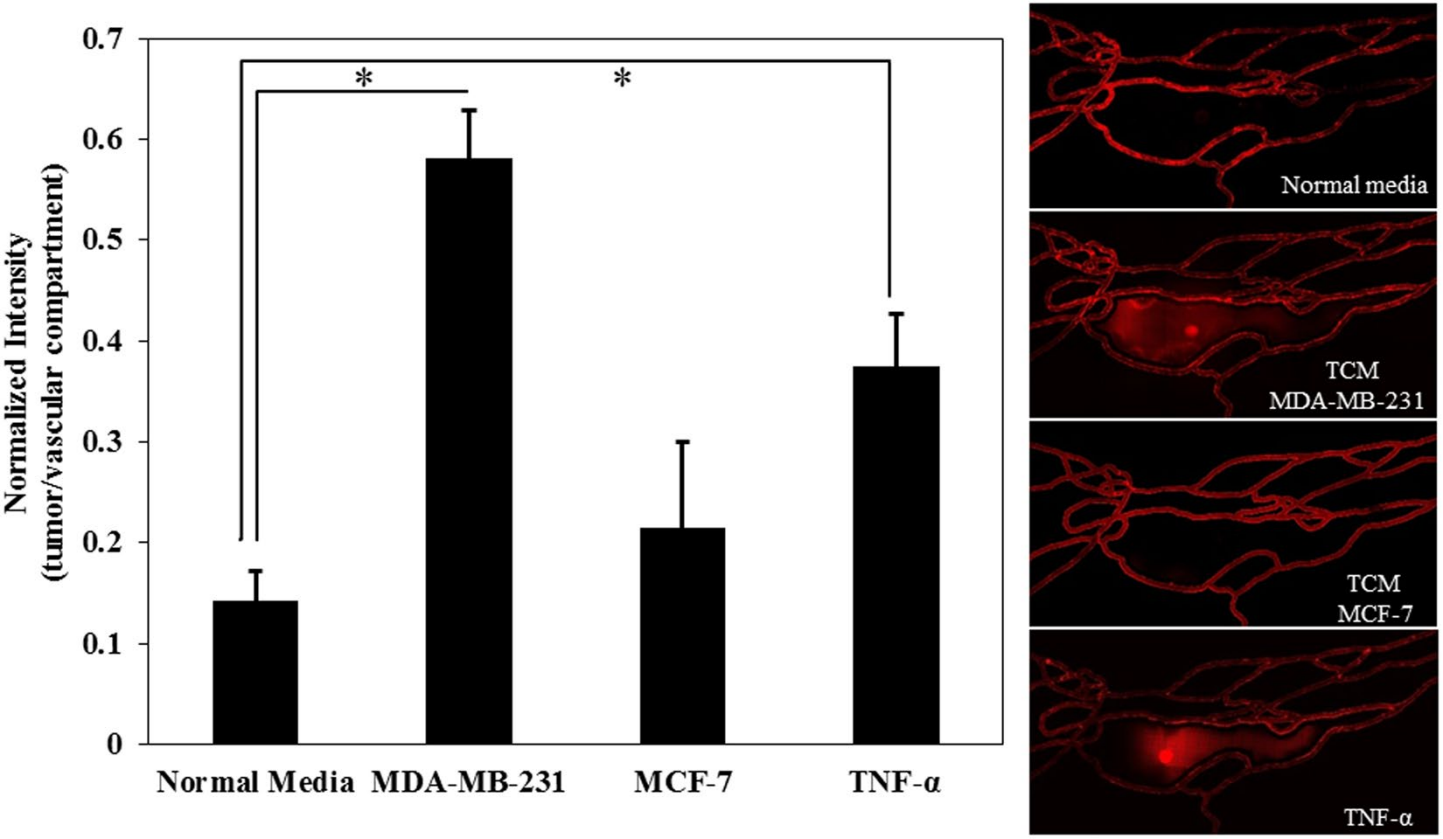
Tumor conditioned media (TCM) (48 h treatment) from highly metastatic MDA-MB-231 tumor cells increased liposome extravasation into the tumor compartment as indicated by the ratio of fluorescent liposome intensity in the tumor compartment to the vascular compartment (panel A). Liposomes extravasated more after MDA-MB-231 conditioned media or TNF-α treatment, but were not affected by TCM obtained from non-metastatic MCF-7 tumor cells (panels B–E). Data are presented as mean ± SEM (n = 3). *Significant difference by *ANOVA*.

In the previous section we have shown that the permeability of HBTAEC to small dye molecules increased in the presence of metastatic MDA-MB-231 tumor cells. However, extravasation of much larger liposomal drug carriers from the vascular to the tumor compartment is controlled by several variables including liposome interaction with endothelial membrane, liposome endocytosis, and diffusion^25–29^. Therefore, extravasation of fluorescently labeled liposomes across the endothelium to the tumor compartment after 30 minutes of perfusion under shear flow was measured following HBTAEC treatment with normal media, TCM, or TNF-α. Consistent with our observation of permeability of fluorescent dextran (Fig. 2), liposome extravasation increased when endothelial cells were treated with TCM from MDA-MB-231 but not with MCF-7 (Fig. 5).

**Figure 6:**
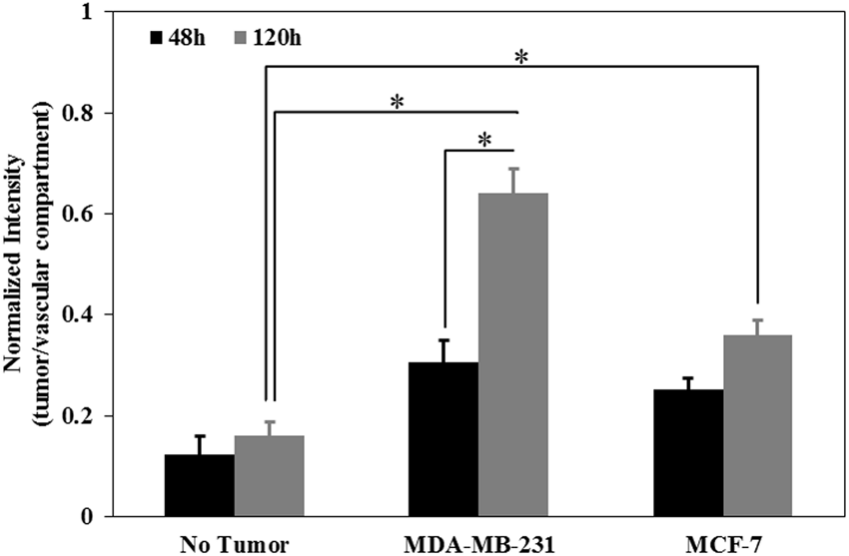
The changes in HBTAEC permeability when co-cultured with tumor cells shown in Fig. 4, resulted in enhanced liposome extravasation with increasing co-culture duration. Data are presented as mean ± SEM (n = 3). *Significant difference by *ANOVA*.

In the bMTM platform, we observed passive accumulation of liposomes which is one of the key features of tumor EPR effect. As shown in Fig. 6, co-culturing either MDA-MB-231 or MCF-7 tumor cells with HBTAEC significantly increased liposome extravasation as compared to HBTAEC cultured without tumor cells. Similar to our observations with fluorescent dextran, increasing the co-culture period from 2 to 5 days further increased liposome extravasation in both tumor co-culture models but the magnitude of increase was higher in the MDA-MB-231 than in the MCF-7 co-culture model.

**Figure 7:**
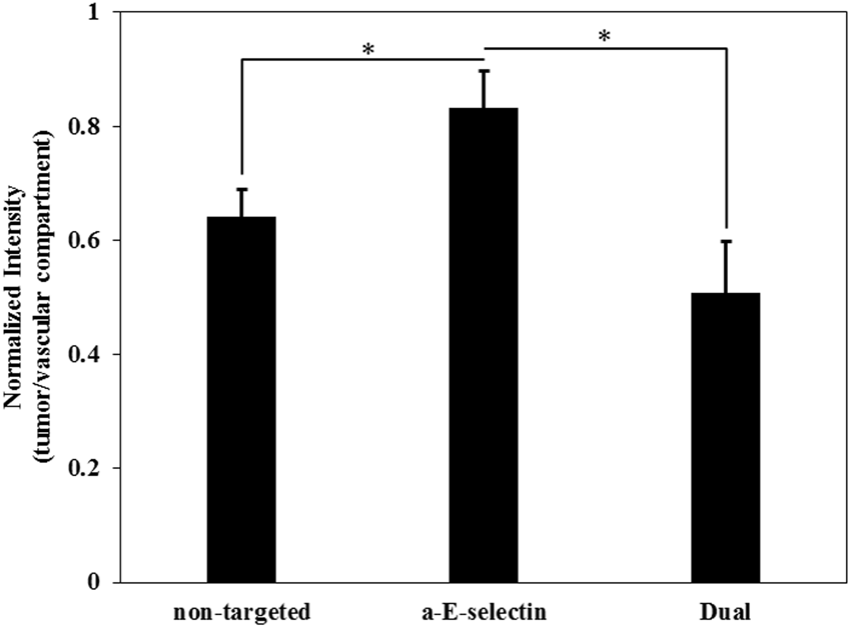
Different formulations of liposomes have different levels of adhesion and extravasation after 48 h of co-culture of HBTAEC and MDA-MB-231 tumor cells. Functionalized liposomes with antibodies to E-selectin (a-E-selectin) have significantly higher adhesion in the vascular compartment and extravasation into the tumor compartment (Figure S3). Dual targeting to both E-selectin and ICAM-1further increased binding when compared to E-selectin single targeting (positive control, Figure S3). Extravasation of E-selectin targeted liposomes was significantly increased when compared to non-targeted liposomes, but no significant difference in extravasation was observed between single and dual targeted liposomes. Data are presented as mean ± SEM (n = 3). *Significant difference by *ANOVA*.

Previously we and others have shown that antibody targeting can significantly increase liposome binding to endothelial cells^30, 31^. As shown in Figs 2–4, the presence of MDA-MB-231 tumor cells changed HBTAEC phenotype and increased its permeability. We therefore tested the hypothesis that functionalizing liposomes will increase their adhesion to the vascular compartment and enhance their permeation into the tumor compartment. To test this hypothesis, we quantified the extravasation of non-targeted, single targeted (a-E-selectin) and dual targeted (50/50 of a-E-selectin and a-ICAM-1) liposomes in the bMTM containing a co-culture of MDA-MB-231 tumor cells and HBTAEC. Antibody targeting significantly increased liposome binding to HBTAECs compared to non-targeted liposomes (Figure S3). Furthermore, dual targeting to both E-selectin and ICAM-1 further increased binding compared to control (Figure S3). Extravasation of E-selectin targeted liposomes was significantly increased when compared to non-targeted liposomes, but no significant difference in extravasation was observed between single and dual targeted liposomes (Fig. 7).

### Immunostainings reveals impaired endothelial cell-cell junctions in the presence of metastatic breast tumor cells

**Figure 8:**
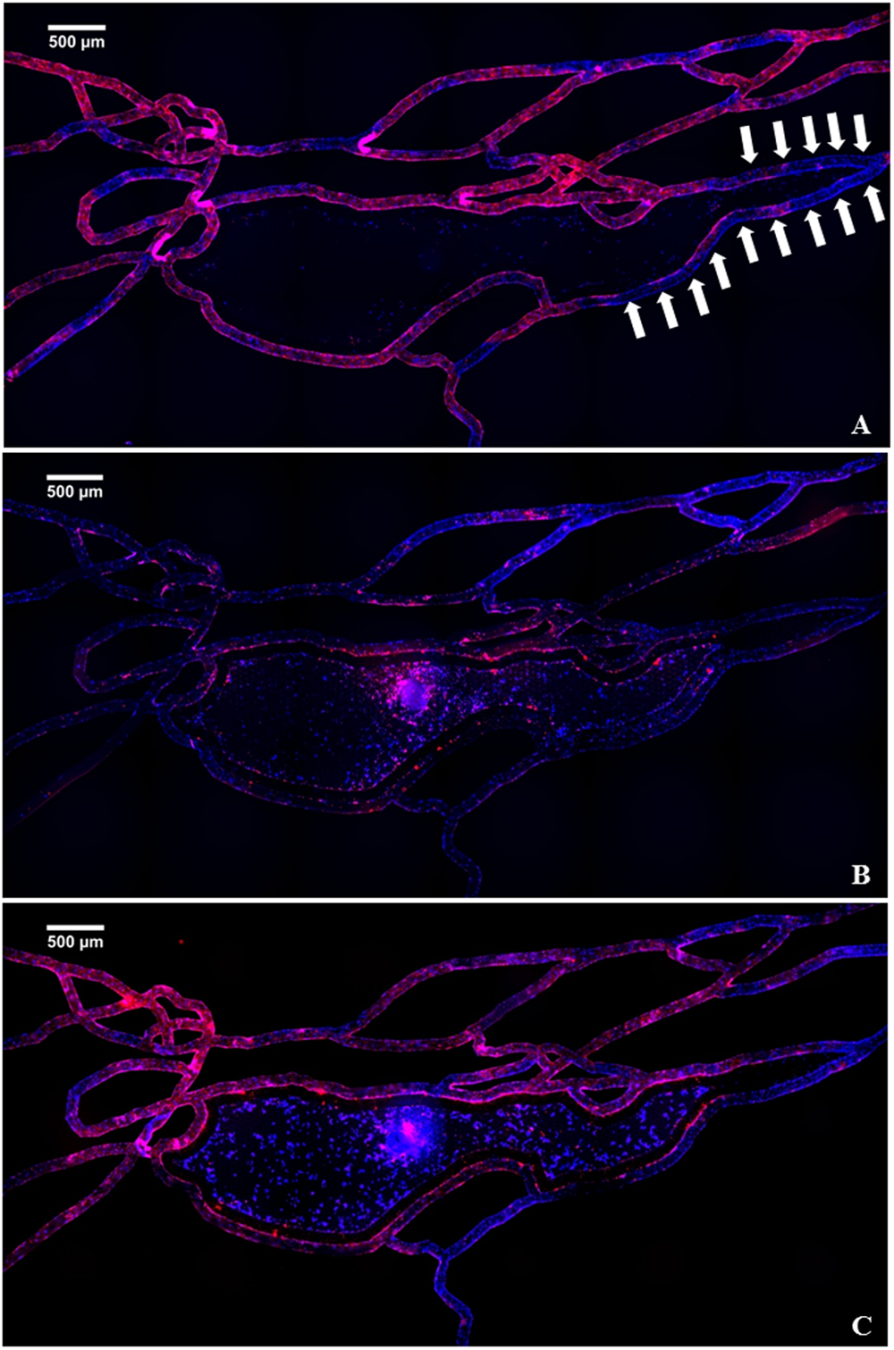
HBTAEC lumen formation as indicated by VE-cadherin (red) is shear flow dependent. Cell nucleus were stained with Hoechst 33342 (blue). At low shear regions (white arrows), the red staining were intermittent indicating less VE-cadherin formation (panel A). When co-cultured with highly metastatic MDA-MB-231 tumor cells, VE-cadherin expression in HBTEAC were inhibted (panel B). The presence of less metastatic MCF-7 tumor cells did not change the formation of adherens junctions (panel C).

Using computational modeling (Figure S1) and experimental validation^24^, we determined that shear rate distribution in the bMTM ranges from 0–90 s^−1^ which matches reported values obtained from *in vivo* tumor vasculature^32, 33^. When cultured without tumor cells, HBTAEC form continuous intercellular adherens junctions under shear flow as indicated by strong continuous VE-cadherin staining in the vascular compartment (Fig. 8A). This shows that our bMTM model not only provides an *in vivo*-like shear flow environment, but also permits junction formation in endothelial cells cultured in the vascular compartment. At very low shear regions (shear rate < 10 s^−1^, indicated by arrows in Fig. 8A), the VE-cadherin formation were intermittent indicating shear flow is critical to junction formation of endothelial cells. In the presence of tumor cells in the tumor compartment, the formation of adherens junctions between endothelial cells were reduced. This effect was more prominent when HBTAECs were co-cultured with metastatic MDA-MB-231 tumor cells (Fig. 8B) than when they were co-cultured with non-metastatic MCF-7 tumor cells (Fig. 8C).

**Figure 9:**
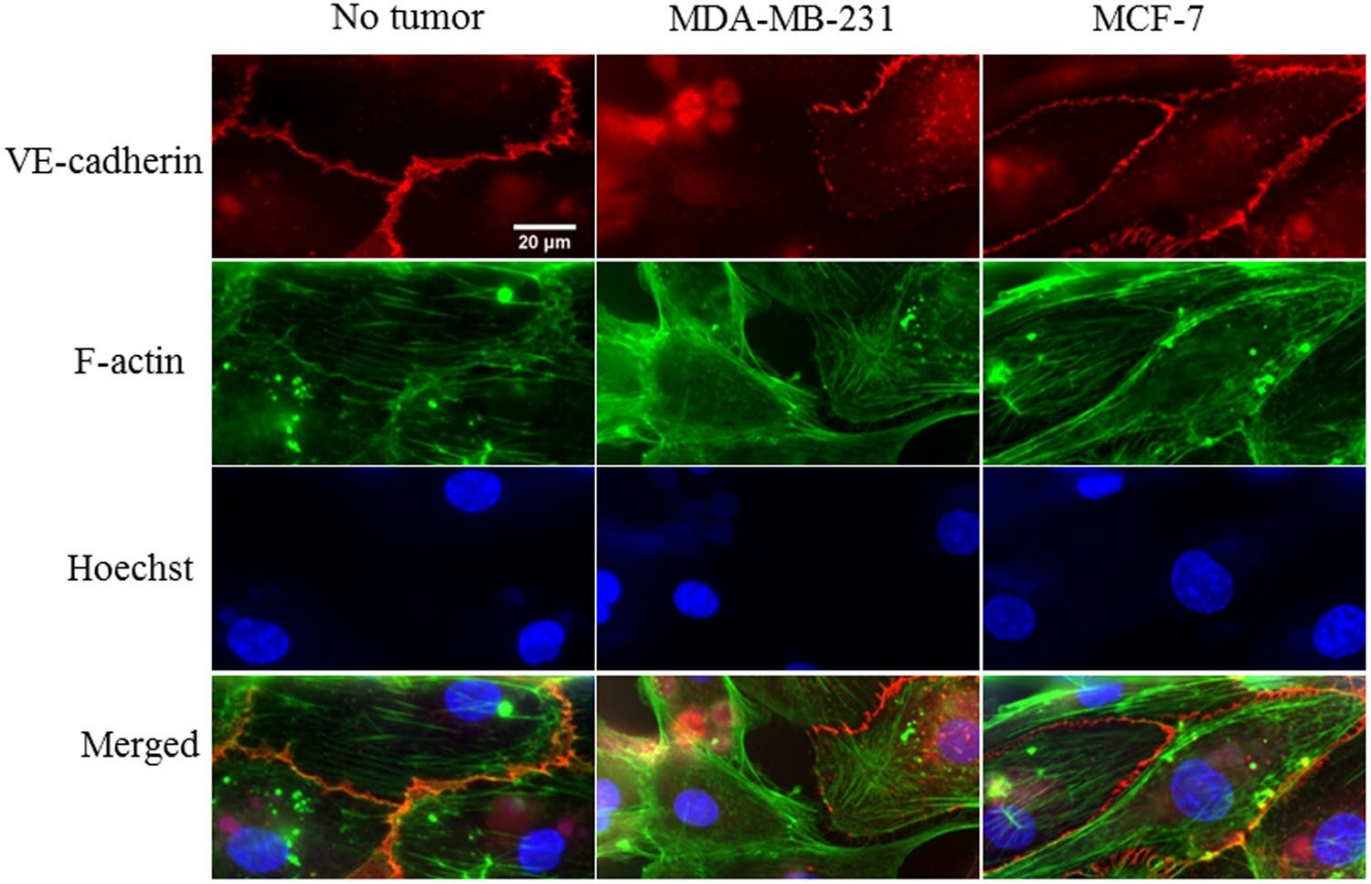
Highly metastatic MDA-MB-231 tumor cells reduced endothelial cell adherens junction (VE-cadherin) molecule expression. When cultured without the presence of tumor cells, adherens junctions were fully established between adjacent cells. When co-cultured with MDA-MB-231 tumor cells, VE-cadherin was partially expressed between adjacent cells, while no change in VE-cadherin expression was observed when HBTAEC were co-cultured with MCF-7 tumor cells. Cell adherens junction molecule was stained with VE-cadherein antibody (red); cell F-actin microfilament was stained with phalloidin (green); cell nucleus was counterstained with Hoechst 33342 (blue).

Adherens junctions and tight junctions are important for regulating the tightness of endothelium barrier^34^. Metastatic MDA-MB-231 cells clearly downregulated adherens junction expression as indicated by the disappearance of VE-cadherin in some cells as well as intermittent VE-cadherin expression along the edges of the remaining cells (Fig. 9). The expression of VE-cadherin in HBTAEC co-cultured with non-metastatic MCF-7 tumor cells showed minimum changes when compared to HBTAEC cultured without tumor cells (Fig. 9).

**Figure 10:**
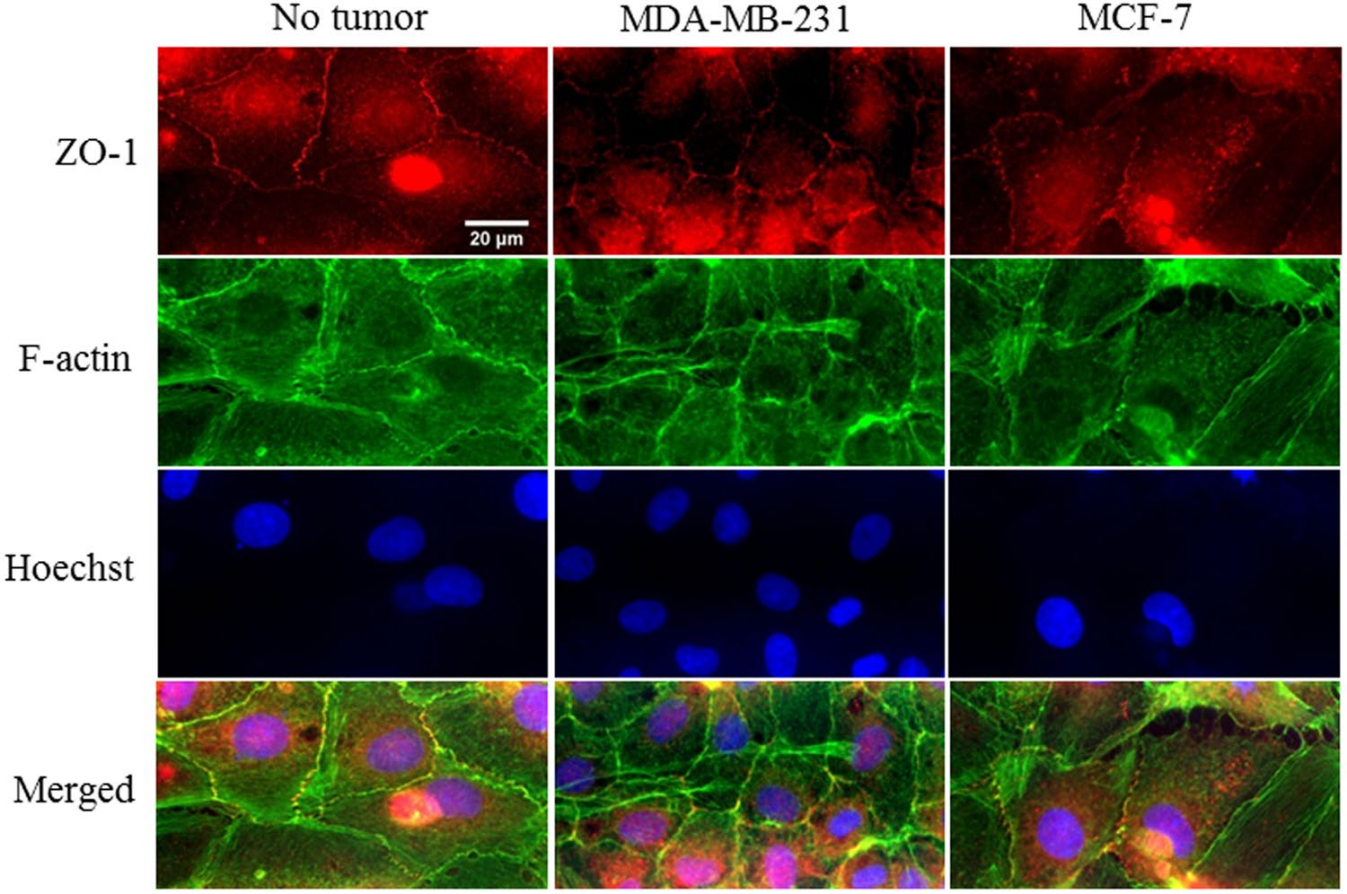
Highly metastatic MDA-MB-231 tumor cells reduced endothelial cell tight junction (ZO-1) molecule expression. When cultured without tumor cells, tight junctions were fully established between adjacent cells. When co-cultured with MDA-MB-231 tumor cells, tight junctions were intermittently expressed along the edge of contacting endothelial cells, while no change was observed when HBTAEC were co-cultured with MCF-7 tumor cells. Cell tight junction molecule was stained with ZO-1 antibody (red); cell F-actin microfilament was stained with phalloidin (green); cell nucleus was counterstained with Hoechst 33342 (blue).

HTBAEC tight junction molecule expression without tumor cell, with MDA-MB-231, or with MCF-7 co-culture was identified by ZO-1 immunostaining (Fig. 10). When cultured without tumor cells, HBTAECs exhibit homogeneously distributed ZO-1 molecule expression. With the presence of the MDA-MB-231 cells, the ZO-1 formation was only detectable at a few spots between contacting endothelial cells. When HBTAEC were co-cultured with non-metastatic MCF-7 cells, ZO-1 molecule expression was not affected. Altogether, these results demonstrate that presence of metastatic tumor cells modulate cell-cell communication as well as endothelial cell barrier function which has significant implications for developing targeted drug delivery strategies.

## Discussion

*In vitro* tumor-endothelial cell co-culture models that closely mimic the *in vivo* tumor microenvironment are valuable tools for understanding heterogeneity and complexity of the tumor microenvironment and for the screening of novel therapeutics. Existing microfluidic platforms have a number of important limitations and for the most part do not mimic the interaction between tumor and endothelial cells under flow^20–22^. Therefore, representation of suitable biomimetic microfluidic tumor microenvironment *in vitro* is of particular importance since there is a lack of adequate research tools for real-time observation and studying of dynamic variation of the cellular characteristics and complex diffusion and extravasation phenomena of novel therapeutics.

A low vessel density is a common feature of malignant tumors and the expansion of endothelial cells during tumor angiogenesis is accompanied by an increase of vessel diameter compared to that in normal tissue^35^. For example, investigation to the microvascular structure of small lung metastases in rats suggested that tumor vessels ranged from 18 to 120 μm in diameter^36^. Other studies have also highlighted the varying size ranges of tumor blood vessels from 8–220 µm^37^. The diameter (100 µM) of the vessels in our bMTM is towards the upper end of what has been found *in vivo* and was selected to demonstrate that our system allows for real-time monitoring of tumor-endothelium interactions. Previously we have shown that the results obtained using this diameter is similar to what has been observed *in vivo* ^38^.

The present study demonstrates an *in vitro* tumor-endothelium interaction model that features an *in vivo*-like microvascular network and simulates the EPR effect. This design represents significant improvements over the traditional static spheroids model and is able to mimic enhanced permeability and retention (EPR) effect observed *in vivo*. In contrast to other microfluidics based tumor platforms, the bMTM design includes a tumor compartment enclosed by endothelial cells grown in a complex microvascular network that is mapped from living tissue. The tumor and vascular compartments are separated via a micro-pillar array interface which allows direct communication of tumor and endothelial cells. The three-dimensional geometry of vascular network addresses the important challenge of studying tumor drug delivery *in vitro* under shear flow conditions which have been shown to be critical in the formation of endothelial cell barrier functions. Furthermore, the optically clear microfluidic platform, and the architecture of the bMTM, allows for visualization and real-time measurements of the dynamic interactions between tumor and endothelial cells.

Human breast cancer associated endothelial cells (HBTAEC), grown on the fibronectin/gelatin coated inner surfaces of vascular compartment of the bMTM form continuous endothelial lumen (Fig. 1, Video S1), thus mimicking the tubular morphology of the *in vivo* microvessels. Tumor-endothelium interactions are critical during cancer metastasis. The ability of our bMTM to reproduce the tubular morphology of the *in vivo* endothelium represents a significant advancement in our efforts to model the *in vivo* tumor microenvironment as compared to the often - used endothelial monolayer models^21, 39^.

Highly metastatic tumors are known to be able to disrupt endothelial junctions and therefore increase vessel permeability^40^. In our study the presence of MDA-MB-231 impaired endothelial cell barrier function as indicated by both permeability and immunostaining assays against VE-cadherin and ZO-1. VE-cadherin is required for forming a tight vascular barrier and its downregulation induces endothelial barrier leak^41^. Tight junction protein occludin interacts with ZO-1 in cytoplasm and stabilizes endothelial barrier function by regulating its tightness^42^. In bMTM, the permeability of endothelial cells to both small dye molecule Texas Red dextran and large liposome drug carrier were increased after co-culturing with MDA-MB-231. Immunostaining indicated impaired cell-cell communication as evident by disrupted VE-cadherin and ZO-1 staining. In contrast to MDA-MB-231 cells, MCF-7 breast cancer cells did not significantly change endothelial cell morphology or permeability, which we hypothesize to be due to the high estrogen dependence of MCF-7 cells. Although tumorigenic in nude mice, MCF-7 is known to be dependent on estrogen supply to grow and metastasize. In agreement with *in vivo* observations^23, 43^ our results indicate that highly metastatic tumor MDA-MB-231 is capable of regulating VE-cadherin (Fig. 9) and tight junction (Fig. 10) expression in endothelial cells.

**Figure 11:**
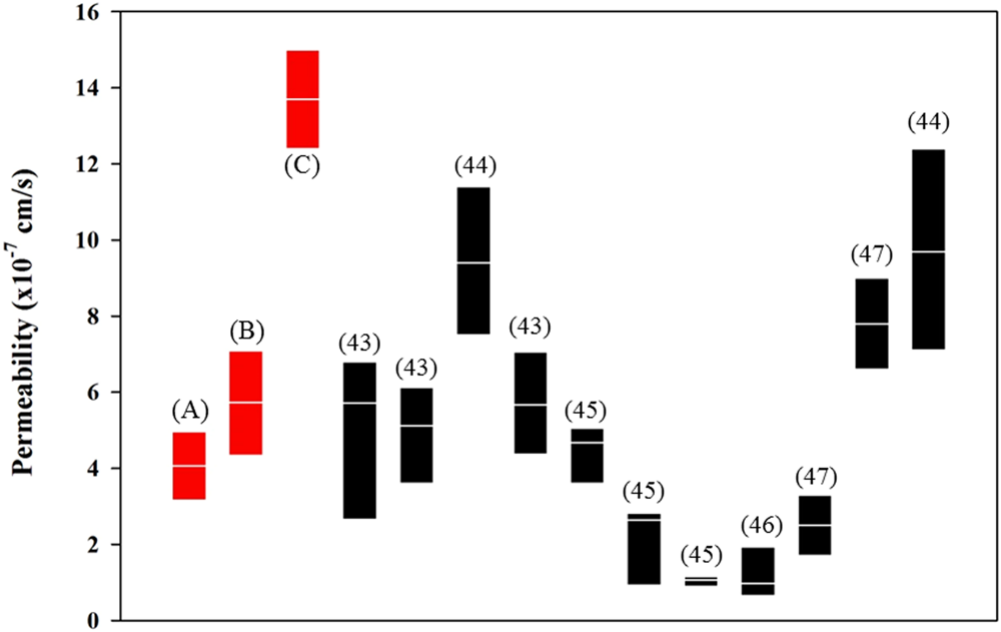
Permeability values obtained in the bMTM with or without tumor cells (in red) are similar to reported *in vivo* values. Experimental results obtained using the bMTM platform (range and median) are highlighted in red and represent permeability of HBTAEC when cultured without tumor cells (**A**), co-cultured with MCF-7 (**B**), or co-cultured with MDA-MB-231 (**C**). The references for each set of *in vivo* data are given in parenthesis.

As shown in Fig. 11, the barrier characteristics of HBTAEC obtained on the bMTM were compared with those reported in the literature using small animal tumor models. Endothelial cell permeability in bMTM was found to be in the range of reported *in vivo* values^44–48^ obtained from similar tumor models in animals (Fig. 11), as well as computationally predicted values^49^, indicating that the magnitude of the EPR effect in the bMTM is similar to those observed *in vivo*. Furthermore, the dynamic processes of drug and drug carrier permeation from vasculature into the tumor can be captured in real time using the bMTM which is a significant advantage as compared to other microfluidics based *in vitro* tumor microenvironment models^20, 22, 50^. Thus, the bMTM platform provides a valuable *in vitro* representation of the EPR effect as observed in the *in vivo* tumor microenvironment.

### Future Work

The main goal of the current study is to establish the feasibility of the bMTM for simulating the EPR effect and characterizing interaction of liposomal drug carriers with tumor endothelium. In the future, we plan to use bMTM to further study key parameters that determine tumor-endothelium interaction, barrier permeability, and tumor drug delivery. Parametric studies can be easily carried out in the bMTM platform to elucidate the effect of many factors on tumor-endothelium interactions including flow rates and molecule sizes on permeability, different ratios of TCM mixture on barrier characteristics, and different targeting moieties and drug carrier formulations on liposome extravasation.

## Conclusion

We have developed a biomimetic microfluidic tumor microenvironment *in vitro* model incorporating co-culture of tumor associated endothelial cells and tumor cells. The design of bMTM not only allows for culturing of endothelial cells under shear flow in realistic complex three-dimensional vascular channels that mimic the microvessel network *in vivo*, but also allows interactions between the endothelial cells and tumor cells as indicated by the successful establishment of tumor EPR effect. The architecture of vascular and tumor compartment fabricated using optically clear polymer and glass combination allows for real-time monitoring of the dynamic processes taking place in the heterogeneous and complex tumor microenvironment. The bMTM platform will enable us to develop rapid screening system for testing the specific response of novel therapeutics in a system that mimics human tissue.

## Materials and Methods

### Microfabrication of the bMTM

This novel bMTM platform consists of cultured tumor derived endothelial cells forming a complete lumen in communication with 3D solid tumors (Fig. 1F–I, Video S1). The microvascular network of the bMTM were designed based on our image database of *in vivo* networks collected according to the ANET system described previously^51–53^. These images were modified to include a tumor compartment in communication with the vascular channels through 2 µm pores, typical pore size found in MCa-IV mouse mammary carcinomas vessel walls^54^. A cylindrical micro-pillar array with prescribed dimensions of 50 µm diameter, 100 µm height and 50 µm spacing was designed to create a scaffold for 3D tumor growth in the tissue area. The microfluidic platform were fabricated using Polydimethylsiloxane (PDMS) based soft lithography based on our established methods^55^. The schematic of the synthetic tumor network is shown in Fig. 1. The shear rate distribution (Figure S1) as well as perfusion trajectory (Figure S2) of the bMTM have been characterized based on both Computational Fluid Dynamics (CFD) modeling and experimental validation, as reported previously^24^.

### Cell culture

Primary human breast tumor associated endothelial cell (HBTAEC, Cell Biologics) was cultured in the vascular compartment of the bMTM to realistically represent the tumor microenvironment. Both MDA-MB-231 and MCF-7 tumor cells obtained from ATCC were cultured in the Dulbecco’s Modified Eagle Medium (DMEM, Invitrogen) supplemented with 10% FBS, 1% Penicillin/Streptomysin. HBTAECs were cultured in Complete Human Endothelial Cell Medium (Cell Biologics). All cells were incubated at 37 °C, 95% humidity with 5% CO_2_ supplement. Confluent cells were sub-cultured at a 1:4 ratio until experiments.

### Establishing the tumor microenvironment

In order to establish a functional endothelium in the bMTM, HBTAECs were seeded and cultured under physiological fluid flow conditions until complete lumen was formed within the vascular channels using our established protocols^56^. Briefly, the bMTM was degassed and washed with sterile deionized (DI) water. The surface of the channels in the vascular compartment was prepared by coating first with fibronectin for 30 minutes at 37 °C and then with gelatin for 5 minutes at 37 °C to facilitate cell attachment. HBTAECs suspended in complete culture media at a concentration of 5 × 10^6^ cells/ml were flowed into the platform using a programmable syringe pump (Harvard Apparatus) and incubated at 37 °C for 4 h prior to flow overnight (0.05 µL/min at the entry of the network). 24 hours after endothelial cell seeding, breast cancer cells (MDA-MB-231 or MCF-7) were seeded into the tumor compartment by suspending the cells in 5:1 ratio of serum free media and Matrigel to support the formation of 3D tumor. The endothelial cells were maintained under above mentioned flow rate, which results in physiological shear values of 0–15 s^−1^ in the bMTM, for 4 days before experiments^57, 58^. Such protocol ensures a complete endothelial cell lumen formation as we have also shown in previous studies for establishing microvascular network on a chip^56, 59^. Devices with incomplete endothelial cell coverage were not used for experiments.

### Endothelial permeability

In order to characterize whether treatment of TCM or the presence of tumor cells in the tumor compartment alters the endothelial cells integrity in the vascular compartment, we quantified permeability of the endothelial cell lining by measuring the flux of 40 kD Texas Red fluorescent dextran (25 μM in HBTAEC culture medium) from the vascular to the tumor compartment. The tumor conditioned media (TCM) was prepared by culturing 10^6^ HBTAECs in 75 cm^2^ culture flask with 12 mL of growth medium for 48 hours, after which the media were collected and filtered as reported in the literature^60–62^. At the time of experiments, the collected TCM was mixed with fresh media at 50/50 ratio and was used as the culture media for endothelial cells as a treatment option.

Following our established protocol^59^, the vascular channel of the bMTM platform was connected to a Hamilton gas tight syringe filled with the dextran solution maintained at 37 °C mounted on a programmable syringe pump (PhD Ultra Syringe pump, Harvard Apparatus). The bMTM was then mounted onto a Nikon TE200 fluorescence microscope (Nikon Instruments) equipped with a temperature controllable automated stage. While the dextran solution was flowing through the channels at a flow rate of 1 μl/min, images were acquired every 1 minute for 3 hours using ORCA Flash 4.0 camera (Hamamatsu Corp.) using NIS Element software (Nikon Instruments).

Permeability of the endothelial cell lining was characterized by the passage of fluorescent dextran from vascular compartment to the tumor compartment over time. Recorded data were analyzed offline using NIS Element software according to our previously reported methods^63^. The following equations were used to calculate permeability (*P*) of dextran across endothelium from the vascular to the tumor compartment:

1 $$P=(1-{H}_{CT})\frac{1}{{I}_{0}}(\frac{dI}{dt})\frac{V}{S}$$

where *H*_*CT*_ is the hematocrit in the vascular channel. Since our media does not include any blood cells, *H*_*CT*_ is set to 0. *I* is the average intensity in the tumor compartment at a given time point, *I*_0_ is the maximum fluorescence intensity in the vascular channel, $\frac{V}{S}$ is the ratio of vascular channel volume to its surface area.

### Drug carrier preparation

Liposomes are U.S. FDA approved spherical lipid bilayer drug delivery vehicles that are biocompatible and biodegradable. Liposomes have been studied and used widely as a nanoscale drug carrier due to its versatility in carrying both hydrophobic and hydrophilic drugs as well as long blood circulation time after pegylation treatment on the surface^64, 65^. In this study, both plain non-targeted liposomes and endothelial adhesion molecule (ICAM-1/E-selectin) targeted liposomes were used to test the effect of passive and active targeting in the bMTM platform. ICAM-1 and E-selectin were shown to be upregulated in tumor induced inflammatory responses^66^, therefore, we expect to see an increased binding of antibody conjugated liposomes to HBTAEC when they are co-cultured with tumor cells.

Plain non-targeted liposomes were composed of 50% hydrogenated soy L-α-phosphatidylcholine (HSPC), 45% cholesterol, 4% 1,2-distearoyl-sn-glycero-3-phosphoethanolamine-N-[(polyethylene glycol)2000]11 (DSPE-PEG2000), and 1% 1,2-dipalmitoyl-*sn*-glycero-3-phosphoethanolamine-N-(lissamine rhodamine B sulfonyl) (ammonium salt) (Rhodamine PE) were prepared by means of solvent evaporation and film formation^67^. Briefly, all four lipid components were dissolved in chloroform (Sigma-Aldrich) which was then removed by nitrogen gas purging at room temperature, followed by overnight evaporation in a Labconco Freezone 1 freeze-dryer. The resulting lipid film was hydrated with phosphate buffered saline (PBS) (Sigma-Aldrich) by gentle mixing resulting in spontaneously organized multilamellar vesicles (MLV). The MLVs were extruded 11 times each through 200 nm & 100 nm pore sized Whatman polycarbonate membranes (GE Healthcare) using a Lipex^TM^ Extruder (Northern Lipids Inc.) to form small unilamellar vesicles (SUV). All lipids were obtained from Avanti Polar Lipids, Inc. All calculations were based on mole ratio.

PEG-maleimide was added to the non-targeted liposome composition for antibody conjugation. Thus the lipid composition became 50% hydrogenated soy L-α-phosphatidylcholine (HSPC), 45% cholesterol, 2% 1,2-distearoyl-sn-glycero-3-phosphoethanolamine-N-[(polyethylene glycol)2000]11 (DSPE-PEG2000), 2% 1,2-distearoyl-*sn*-glycero-3-phosphoethanolamine-N-[maleimide(polyethylene glycol)-2000] (ammonium salt) (DSPE-PEG-maleimide) and 1% 1,2-dipalmitoyl-sn-glycero-3-phosphoethanolamine-N-(lissamine rhodamine B sulfonyl) (ammonium salt) (Rhodamine PE). Lipids were mixed, dried, hydrated, and extruded as described in the previous section.

Functionalized targeted immunoliposomes were produced thereafter by conjugating their surfaces with different ratios of antibodies against adhesion molecules ICAM-1 (a-ICAM-1) or E-selectin (a-E-selectin) using 2-iminothiolane linker. Mouse monoclonal anti human E-selectin/ICAM-1/dual antibody (Santa Cruz biotechnology) was attached to the distal end of PEG chains on liposome surface to form immunoliposomes. The antibody was first thiolated with 2-iminothiolane (Sigma-Aldrich) at pH 8.0. The introduced thiol groups were then coupled with maleimide groups on DSPE-PEG2000 component of the liposomes at pH 6.5. Unconjugated antibodies were removed by Sepharose CL-4B gravity column. Detailed liposome preparation and characterization procedures can be found in our previous publications^30, 67–70^.

### Drug carrier binding and extravasation

Liposome extravasation is regulated in part by the EPR effect. Similar to the permeability experiment, liposome extravasation from the vascular channels in intact or leaky configuration to the tumor compartment was quantified using imaging methods as described above in section 4. The vascular channels of the bMTM were perfused with fluorescent liposomes suspended in culture media for 30 minutes at a flow rate of 1 μl/min at 37 °C. The bMTM was then washed with PBS buffer 3X to remove unbound liposomes. Images of the bMTM were taken using the same camera and microscope as described in section 4. Intensity of liposomes in both vascular and tumor compartments were then compared using ImageJ software.

### Immunofluorescence staining

In order to examine endothelial cell barrier function after co-culture with tumor cells in the bMTM, the formation of endothelial cell-to-cell adherens junctions and tight junctions was characterized using immunostaining against VE-cadherin and zonula occludens (ZO-1) respectively. Briefly, the bMTM was perfused with 4% natural buffered formalin (Sigma-Aldrich) to fix the cells followed by 10 minutes treatment with 0.1% Triton X-100 (Sigma-Aldrich) to expose VE-cadherin/ZO-1 protein. After blocking with 5% goat serum in PBS for 1 hour at 37 °C, mouse monoclonal primary antibody (Santa Cruz biotechnology) against VE-cadherin/ZO-1 (1:100) was flowed through the vascular channel and the bMTM was incubated overnight at 4 °C. In the second day, fluorophore-conjugated secondary antibodies Alexa fluor 594 goat anti-mouse IgG (1:400, Santa Cruz biotechnology) were flowed and the bMTM was then incubated for 1 hour at 37 °C. Cells were washed with PBS containing 5% serum between each step using syringe. Images were taken using the same microscope and camera system as described before.

### Data analysis

Data are presented as Mean ± SEM, and *Analysis of Variance (ANOVA)* with Bonferroni post-hoc was applied to obtain statistical significance. Significance levels were set at α = 0.05.

## Electronic supplementary material

supplementary data

supplementary video
