# Supplementary material for: A Biomimetic Microfluidic Tumor Microenvironment Platform Mimicking the EPR Effect for Rapid Screening of Drug Delivery Systems
Source: Sci Rep. 2017 Aug 24;7:9359. doi: 10.1038/s41598-017-09815-9 (PMC5571192; doi:10.1038/s41598-017-09815-9)
Supplement: Supplementary file 1 — supplementary data [file 41598_2017_9815_MOESM1_ESM.doc]

Title: A Biomimetic Microfluidic Tumor Microenvironment Platform Mimicking the EPR Effect for Rapid Screening of Drug Delivery Systems

Author affiliation:

Yuan Tang^1^, Fariborz Soroush^1^, Joel B. Sheffield^2^, Bin Wang^1,3^, Balabhaskar Prabhakarpandian^4^ Mohammad F. Kiani^1,5^

^1^Department of Mechanical Engineering, Temple University, Philadelphia, PA 19122 USA

^2^Department of Biology, Temple University, Philadelphia, PA 19122 USA

^3^Department of Biomedical Engineering, Widener University, Chester, PA 19013 USA

^4^Biomedical Technology, CFD Research Corporation, Huntsville, AL 35806 USA

^5^Department of Radiation Oncology, Lewis Katz School of Medicine, Temple University

Philadelphia, PA 19140 USA

*Corresponding author:*

Name: Mohammad F. Kiani

Address: 1947 N.12^th^ St, Philadelphia, PA 19122

Phone: 215-204-4644

Email: mkiani@temple.edu

Supplement Data:

**Shear Rate (s^-1^)**


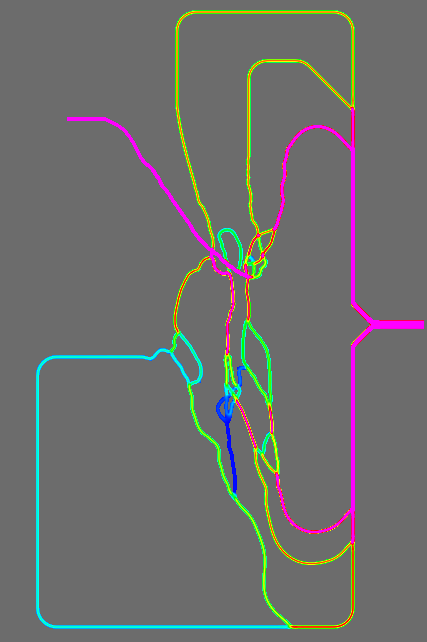


**0**


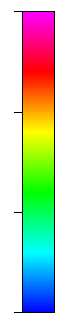


**30**

**60**

**90**

Figure S1. Shear distribution in the channels of bMTM.

Figure S2. Trajectory of FITC solution perfusion from inlet to outlet in bMTM. Top panels: computational fluid dynamics (CFD) predicted perfusion profiles. Bottom panels: experimentally measured perfusion profiles. Images are pseudo-colored to show relative concentration gradients. The scale is normalized unit with blue (no perfusion; 0) and magenta (complete perfusion; 1).


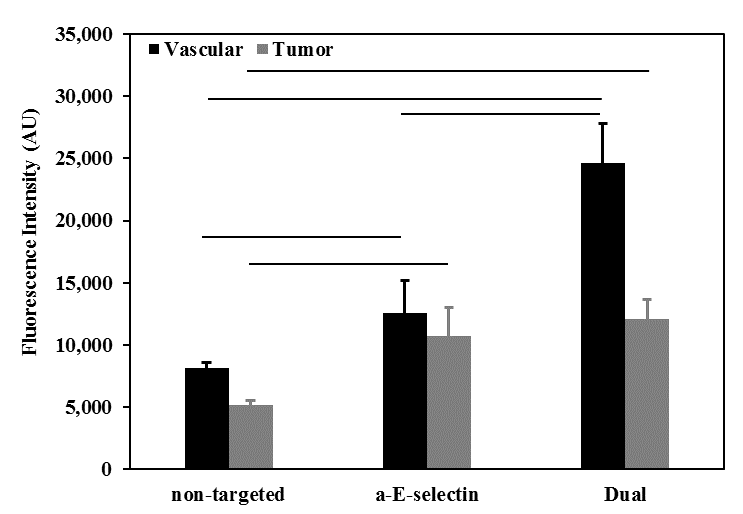


*

*

*

*

*

Figure S3. Antibody targeting significantly increased liposome binding to HBTAECs compared to non-targeted liposomes. Dual targeting to both E-selectin and ICAM-1further increased binding when compared to E-selectin single targeting. Extravasation of E-selectin targeted liposomes was significantly increased when compared to non-targeted liposomes, but as shown in Figure 7, no significant difference in extravasation was observed between single and dual targeted liposomes. HBTAEC and MDA-MB-231 tumor cells were co-cultured for 48 h. Data are presented as mean ± SEM (n = 3). *Significant difference by two-way *ANOVA*.
